# Supplementary material for: Public policy and economic dynamics of COVID-19 spread: A mathematical modeling study
Source: PLoS One. 2020 Dec 22;15(12):e0244174. doi: 10.1371/journal.pone.0244174 (PMC7755180; doi:10.1371/journal.pone.0244174)
Supplement: S1 Appendix — This file contains the complete description of our model and the system of differential equations used in our model. (PDF) [file pone.0244174.s001.pdf]

S1 Appendix. Complete model formulation

We simulate disease spread for 76 weeks, an approximation of time to vaccine, in a population of 330 million individuals, similar to that of the United States.

Our model consists of two interconnected sub-models, an epidemiological model and an economic model. The epidemiological model expands the classical SEIR model and includes several aspects that shape the spread of COVID-19, e.g., the difference in mortality according to age, and the effect of isolation restrictions. The economic model simulates the impact of isolation restrictions on the economic productivity of the population. While isolation restrictions control the spread of the disease, they have a detrimental effect on the economy, which in turn, pushes policymakers to lift those restrictions. We combine the epidemiological and economic effects of isolation policies to explore policies that balance these effects. Our model is described in detail below.

Epidemiological model

The compartmental model graphically described in the model overview section is described by the system of differential equations presented below. The system of equations is duplicated for the senior population but using different parameter values. This change is reflected by the subscript $i = G, S$ in the variables of the model, which indicates whether the value is for the general population or the senior population.

$$\frac{dS_i}{dt} = -\beta \frac{I_G + I_S}{N} S_i - \epsilon \frac{H_G + H_S}{N} S_i + \kappa_i Iso_i^S - \boldsymbol{\theta}_i^T \cdot \boldsymbol{v}_i S_i \tag{1}$$

$$\frac{dIso_i^S}{dt} = -\kappa_i Iso_i^S + \boldsymbol{\theta}_i^T \cdot \boldsymbol{v}_i S_i \tag{2}$$

$$\frac{dE_i}{dt} = \beta \frac{I_G + I_S}{N} S_i + \epsilon \frac{H_G + H_S}{N} S_i - \gamma_i E_i + \kappa_i Iso_i^E - \boldsymbol{\theta}_i^T \cdot \boldsymbol{v}_i E_i \tag{3}$$

$$\frac{dIso_i^E}{dt} = -\gamma_i Iso_i^E - \kappa_i Iso_i^E + \boldsymbol{\theta}_i^T \cdot \boldsymbol{v}_i E_i \tag{4}$$

$$\frac{dI_i}{dt} = \gamma_i E_i - (1 - p_i)\nu_i I_i - p_i \chi_i I_i + \kappa_i Iso_i^I - \boldsymbol{\theta}_i^T \cdot \boldsymbol{v}_i I_i \tag{5}$$

$$\frac{dIso_i^I}{dt} = \gamma_i Iso_i^E - (1 - p_i)\nu_i Iso_i^I - p_i \chi_i Iso_i^I - \kappa_i Iso_i^I + \boldsymbol{\theta}_i^T \cdot \boldsymbol{v}_i I_i \tag{6}$$

$$\frac{dH_i}{dt} = -\delta_i (HFR_i) H_i - \rho_i (1 - HFR_i) H_i + p_i \chi_i I_i + p_i \chi_i Iso_i^I \tag{7}$$

$$\frac{dD_i}{dt} = \delta_i (HFR_i) H_i \tag{8}$$

$$\frac{dR_i}{dt} = (1-p_i)\nu_i I_i + \rho_i(1-HFR_i)H_i + \kappa_i Iso_i^R - \boldsymbol{\theta}_i^T \cdot \boldsymbol{v}_i R_i \tag{9}$$

$$\frac{dIso_i^R}{dt} = (1-p_i)\nu_i Iso_i^I - \kappa_i Iso_i^R + \boldsymbol{\theta}_i^T \cdot \boldsymbol{v}_i R_i \tag{10}$$

$$\text{HFR}_i = \begin{cases} \text{Saturation factor} \cdot \left[\frac{365-t}{365}(\text{HFR}_{\text{init}_\text{i}} - \text{HFR}_{\text{final}_\text{i}}) + \text{HFR}_{\text{final}_\text{i}}\right], & \text{if } t \leq 365 \\ \text{Saturation factor} \cdot \text{HFR}_{\text{final}_\text{i}}, & \text{otherwise} \end{cases} \tag{11}$$

In this model, the susceptible population gets exposed to the virus at a rate proportional to the fraction of infected asymptomatic and hospitalized individuals in the population. β and ϵ regulate the contagiousness of the infected asymptomatic and hospitalized individuals, respectively. The subscript S represents the fraction of a given compartment that represents the senior population. N is the total population.

The public policy measures that drive the population into isolation are reflected by the κ and $\boldsymbol{\theta}^T \cdot \boldsymbol{v}$ terms. κ determines the rate at which the non-hospitalized population leaves isolation compartments. $\boldsymbol{v}$ is a column vector containing the number of infected people since the beginning of the outbreak, the number of deaths since the beginning of the outbreak, the number of infected people in the previous 7 days, the number of deaths in the previous 7 days, and the current state of the economy expressed as the net productivity change since the beginning of the outbreak. $\boldsymbol{\theta}$ is a weight vector that reflects the relative importance the public policy makers pay to the different elements in $\boldsymbol{v}$. In mathematical notation this is expressed as:

$$\boldsymbol{\theta}^T \boldsymbol{v} = [w_1 \; w_2 \; w_3 \; w_4 \; w_5\,] \begin{bmatrix} \text{total number of infected people} \\ \text{total number of deaths} \\ \text{number of infected people in the previous 7 days} \\ \text{number of deaths in the previous 7 days} \\ \text{current state of the economy} \end{bmatrix}$$

The infected asymptomatic individuals require hospitalization with probability p, reflected by the forking of the compartments containing infected and isolated infected individuals.

The HFR decreases linearly with time to reflect the increasing knowledge and experience of the healthcare system. The HFR is also modified based on hospital resource strain or saturation. We define the point of hospital resource strain as the point were the number of infected individuals that require hospitalization reaches the number of unoccupied hospital beds. Similarly, we define hospital saturation as the point where the number of individuals that needs

hospitalization reaches the total number of hospital beds. When the hospital resources are strained, the HFR is multiplied by 1.5, and by 2 if the hospital resources are saturated. This is expressed by the saturation factor.

Economic Model

We categorize the population into four groups: older workers who have higher ability to work in an isolated state ($y_{\mathrm{S,H}}$), senior workers who have lower ability to work in an isolated state ($y_{\mathrm{S,L}}$), non-senior workers who have higher ability to work in an isolated state ($y_{\mathrm{G,H}}$), and non-senior workers who have lower ability to work in an isolated state ($y_{\mathrm{G,L}}$). The workers who have lower ability to work in isolated state lose a higher percentage of their productivity (represented by ψ) than workers who have higher ability to work in an isolated state (represented by ϕ). In our simulation, we use college education as a proxy for ability to work in an isolated state, as found by Mongey et al. [1] We find the percentage of each group in the labor force. Although our theoretical model is more flexible, we implement our model using data based on the United States context. For productivity levels of our different types of workers, we use the 2018 American Community Survey, and average wage income for each group. For each group, we look at labor force participation rate, and average wage income conditional on labor force participation. The values $y_{\mathrm{S,H}}$, $y_{\mathrm{S,L}}$, $y_{\mathrm{G,H}}$, $y_{\mathrm{G,L}}$ are calculated as labor force participation rate multiplied by average wage income. We also use the American Community Survey to see the proportion of the four types of workers present in the population.

We use two economic types of workers, i_{high} who have higher ability to work in an isolated state and individuals of type i_{low} who have lower ability to work in an isolated state. Nothing in our model restricts the number of types to two: it can be any finite number.

Total economic output is the sum of the output of individuals in each group subtracted by the costs of treating the infected. For the long-term economic output beyond the pandemic period, labor supply matters, so deaths impose a penalty on future economic output, dependent on the discount rate r. This is modelled as follows:

$$Y = Y_{\mathrm{low}} + Y_{\mathrm{high}} - C_{\mathrm{treatment}} - \frac{Y_{\mathrm{dead}}}{r} \tag{12}$$

The productivity of each labor group is the sum of productivity of all individuals in each labor group.

$$Y_{\mathrm{low}} = \sum y_{\mathrm{il}}. \tag{13}$$

$$Y_{\mathrm{high}} = \sum y_{\mathrm{ih}}. \tag{14}$$

The cost of treating the infected is the number of infected symptomatic of each risk type and the cost of treating each risk type.

$$C_{\text{treatment}} = C_{\text{hr}} \cdot I_{S_{\text{hr}}} + C_{\text{lr}} \cdot I_{S_{\text{lr}}} \tag{15}$$

Different workers of the same labor type might be under different isolation restrictions based on their age or risk type. For each worker occupation type h, l, pre-pandemic productivity is drawn from a distribution $F(h), F(l)$, respectively. In our simulation, we use four levels of productivity: one for each type of worker: $y_{\text{S,H}}$, $y_{\text{S,L}}$, $y_{\text{G,H}}$, and $y_{\text{G,L}}$, as previously described.

For workers of occupation type i_{high}, output in isolation for susceptible (S), exposed (E), or infected asymptomatic (I_A) workers is

$$y_{\text{ih}} = \phi \cdot (1 - t_{\text{h}}) \cdot h_{\text{i}}, \tag{16}$$

where ϕ is the penalty for i_{high} workers in isolation, t_{h} is a linear penalty term for the length of the negative infection shock, and h_{i} is the productivity of the i_{high} type in the pre-pandemic state. If (S), exposed (E), or infected asymptomatic (I_A) workers are not in isolation, output is

$$y_{\text{ih}} = (1 - t_{\text{h}}) \cdot h_{\text{i}}. \tag{17}$$

When workers are hospitalized (I_H), productivity is 0. When workers recover from illness, output is

$$y_{\text{ih}} = (\phi + \iota) \cdot (1 - t_{\text{h}}) \cdot h_{\text{i}} \tag{18}$$

if isolated, and $(1 - t_{\text{h}}) \cdot h_{\text{i}}$ if not isolated, where ι represents the productivity boost in isolation from immunity.

For workers of occupation type i_{low}, output in isolation for susceptible (S), exposed (E), or infected asymptomatic (I_A) workers is

$$y_{\text{il}} = \psi \cdot (1 - t_l) \cdot l_{\text{i}}, \tag{19}$$

where ψ is the penalty for i_{low} workers in isolation, t_{l} is a linear penalty term for the length of the negative infection shock, and l_{i} is the productivity of the i_{low} type in the pre-pandemic state. If (S), exposed (E), or infected asymptomatic (I_A) workers are not in isolation, output is $y_{\text{il}} = (1 - t_{\text{l}}) \cdot l_{\text{i}}$.

When workers are hospitalized (I_H), productivity is 0. When workers recover from illness, output is

$$y_{\text{il}} = (\psi + \xi) \cdot (1 - t_{\text{l}}) \cdot l_{\text{i}} \tag{20}$$

if isolated, and $(1 - t_{\text{l}}) \cdot l_{\text{i}}$ if not isolated, where ξ represents the productivity boost in isolation from immunity.

The dynamics of this economy are as follows:

$$
\begin{aligned}
\frac{dY_{\mathrm{S,H}}}{dt} = \Bigg[& \left(\frac{dIso_{\mathrm{S}}^{S}}{dt} + \frac{dIso_{\mathrm{S}}^{E}}{dt} + \frac{dIso_{\mathrm{S}}^{I}}{dt} \right) \cdot \kappa_{\mathrm{s}} \phi (1 - t_{\mathrm{h}}) \\
& + \left(\frac{dS_{\mathrm{S}}}{dt} + \frac{dE_{\mathrm{S}}}{dt} + \frac{dI_{\mathrm{S}}}{dt} \right) \cdot (\boldsymbol{\theta}_{\mathrm{s}}^{T} \cdot \boldsymbol{v})(1 - t_{\mathrm{h}}) \\
& + \left(\frac{dH_{\mathrm{S}}}{dt} + \frac{dD_{\mathrm{S}}}{dt} \right) \cdot 0 \\
& + \left(\frac{dIso_{\mathrm{S}}^{R}}{dt} \right) \cdot \kappa_{\mathrm{s}} (\phi + \iota)(1 - t_{\mathrm{h}}) \\
& + \left(\frac{dR_{\mathrm{S}}}{dt} \right) \cdot (\boldsymbol{\theta}_{\mathrm{s}}^{T} \cdot \boldsymbol{v})(1 - t_{\mathrm{h}}) \Bigg] Y_{\mathrm{S,H}}
\end{aligned} \tag{21}
$$

$$
\begin{aligned}
\frac{dY_{\mathrm{S,L}}}{dt} = \Bigg[& \left(\frac{dIso_{\mathrm{S}}^{S}}{dt} + \frac{dIso_{\mathrm{S}}^{E}}{dt} + \frac{dIso_{\mathrm{S}}^{I}}{dt} \right) \cdot \kappa_{\mathrm{s}} \psi (1 - t_{\mathrm{l}}) \\
& + \left(\frac{dS_{\mathrm{S}}}{dt} + \frac{dE_{\mathrm{S}}}{dt} + \frac{dI_{\mathrm{S}}}{dt} \right) \cdot (\boldsymbol{\theta}_{\mathrm{s}}^{T} \cdot \boldsymbol{v})(1 - t_{\mathrm{l}}) \\
& + \left(\frac{dH_{\mathrm{S}}}{dt} + \frac{dD_{\mathrm{S}}}{dt} \right) \cdot 0 \\
& + \left(\frac{dIso_{\mathrm{S}}^{R}}{dt} \right) \cdot \kappa_{\mathrm{s}} (\psi + \xi)(1 - t_{\mathrm{l}}) \\
& + \left(\frac{dR_{\mathrm{S}}}{dt} \right) \cdot (\boldsymbol{\theta}_{\mathrm{s}}^{T} \cdot \boldsymbol{v})(1 - t_{\mathrm{l}}) \Bigg] Y_{\mathrm{S,L}}
\end{aligned} \tag{22}
$$

$$
\begin{aligned}
\frac{dY_{\mathrm{G,H}}}{dt} = \Bigg[& \left(\frac{dIso^{S}}{dt} + \frac{dIso^{E}}{dt} + \frac{dIso^{I}}{dt} \right) \cdot \kappa \phi (1 - t_{\mathrm{h}}) \\
& + \left(\frac{dS}{dt} + \frac{dE}{dt} + \frac{dI_{\mathrm{A}}}{dt} \right) \cdot (\boldsymbol{\theta}^{T} \cdot \boldsymbol{v})(1 - t_{\mathrm{h}}) \\
& + \left(\frac{dH}{dt} + \frac{dD}{dt} \right) \cdot 0 \\
& + \left(\frac{dIso^{R}}{dt} \right) \cdot \kappa (\phi + \iota)(1 - t_{\mathrm{h}}) \\
& + \left(\frac{dR}{dt} \right) \cdot (\boldsymbol{\theta}^{T} \cdot \boldsymbol{v})(1 - t_{\mathrm{h}}) \Bigg] Y_{\mathrm{G,H}}
\end{aligned} \tag{23}
$$

$$
\begin{aligned}
\frac{dY_{\mathrm{G,L}}}{dt} = \Bigg[&\left(\frac{dIso^S}{dt} + \frac{dIso^E}{dt} + \frac{dIso^I}{dt} \right) \cdot \kappa\psi(1-t_{\mathrm{l}}) \\
&+ \left(\frac{dS}{dt} + \frac{dE}{dt} + \frac{dI_{\mathrm{A}}}{dt} \right) \cdot (\boldsymbol{\theta}^T \cdot \boldsymbol{v})(1-t_{\mathrm{l}}) \\
&+ \left(\frac{dH}{dt} + \frac{dD}{dt} \right) \cdot 0 \\
&+ \left(\frac{dIso^R}{dt} \right) \cdot \kappa(\psi+\xi)(1-t_{\mathrm{l}}) \\
&+ \left(\frac{dR}{dt} \right) \cdot (\boldsymbol{\theta}^T \cdot \boldsymbol{v})(1-t_{\mathrm{l}}) \Bigg] Y_{\mathrm{G,L}}
\end{aligned} \tag{24}
$$

$$
\frac{dC_{\mathrm{L}}}{dt} = c_{\mathrm{l}} \left(\frac{dH}{dt} \right) \tag{25}
$$

$$
\frac{dC_{\mathrm{D}}}{dt} = c_{\mathrm{d}} \left(\frac{dD}{dt} \frac{Y_{\mathrm{G,H}} + Y_{\mathrm{G,L}}}{POP_{\mathrm{G,H}} + POP_{\mathrm{G,L}}} + \frac{dD_{\mathrm{S}}}{dt} \frac{Y_{\mathrm{S,H}} + Y_{\mathrm{S,L}}}{POP_{\mathrm{S,H}} + POP_{\mathrm{S,L}}} \right) \tag{26}
$$

$$
\frac{dC_{\mathrm{H}}}{dt} = c_{\mathrm{h}} \left(\frac{dH_{\mathrm{S}}}{dt} \right) \tag{27}
$$

where, as before, for i_{high} workers, ϕ is the productivity penalty in isolation, t_{h} is a linear penalty term for the length of the negative infection shock, ι is the represents the productivity boost in isolation from immunity, and for i_{low} workers, ψ is the productivity penalty in isolation, t_{l} is a linear penalty term for the length of the negative infection shock, and ξ is the productivity boost in isolation from immunity. In our simulation, $Y_{\mathrm{S,H}}$ represents the productivity of the older workers who have higher ability to work in an isolated state ($\sum y_{\mathrm{S,H}}$), $Y_{\mathrm{S,L}}$ represents the total productivity of all older workers who have lower ability to work in an isolated state ($\sum y_{\mathrm{S,L}}$), $Y_{\mathrm{G,H}}$ represents the productivity of non-senior workers who have higher ability to work in an isolated state ($\sum y_{\mathrm{G,H}}$), and $Y_{\mathrm{G,L}}$ represents the productivity of non-senior workers who have lower ability to work in an isolated state ($\sum y_{\mathrm{G,L}}$). C_{D} represents the cost to society from dead individuals, C_{H} represents the cost of treating seniors who are hospitalized, and C_{L} represents the cost of treating non-seniors who are hospitalized. $POP_{\mathrm{S,H}}$ represents the number of all older workers who have higher ability to work in an isolated state, $POP_{\mathrm{S,L}}$ represents the number of all older workers who have lower ability to work in an isolated state, $POP_{\mathrm{G,H}}$ represents the number of non-senior workers who have higher ability to work in an isolated state, and $POP_{\mathrm{G,L}}$ represents the number of non-senior workers who have lower ability to work in an isolated state.

Equations (21) - (27) follow directly from equations (12) - (20).

References

[1] Mongey S, Pilossoph L, Weinberg A. Which workers bear the burden of social distancing policies? National Bureau of Economic Research; 2020.
