# Supplementary material for: Public policy and economic dynamics of COVID-19 spread: A mathematical modeling study
Source: PLoS One. 2020 Dec 22;15(12):e0244174. doi: 10.1371/journal.pone.0244174 (PMC7755180; doi:10.1371/journal.pone.0244174)
Supplement: S1 File — This file contains the values of the parameters used in our model and the relevant references. (PDF) [file pone.0244174.s002.pdf]

S1 File

S1 Table: Model parameters.

Parameter	Description	Value	Reference
$\max_{\mathrm{HFR}}$	Hospital fatality rate for non-seniors in week 0	1.5%	[1,2]
$\min_{\mathrm{HFR}}$	Hospital fatality rate for non-seniors in week 76	0.5%	[3,4]
$\max_{\mathrm{HFR_s}}$	Hospital fatality rate for seniors in week 0	12%	[1,2,5]
$\min_{\mathrm{HFR_s}}$	Hospital fatality rate for seniors in week 76	9%	[3,4]
Strain point	Minimum hospitalized to impact HFR through strain	0.08% of population	[6]
Saturation point	Minimum hospitalized to impact HFR through saturation	0.22% of population	[6]
β	average transmission rate for infected	1 [1/days]	[7,8]
ϵ	transmission rate for hospitalized	0.1 [1/days]	[7]
γ	disease onset rate	1/6.4 [1/days]	[7]
$p_{\mathrm{hospitalized}}$	probability of being hospitalized for infected non-seniors	3%	[4]
$p_{\mathrm{hospitalized_s}}$	probability of being hospitalized for infected seniors	15%	[4,5]
χ	transition rate from non-hospitalized to hospitalized for non-seniors	1/7 [1/days]	[9]
χ_S	transition rate from non-hospitalized to hospitalized for seniors	0.1857 [1/days]	[5,9]
δ	defunction rate for hospitalized patients	1/7 [1/days]	[9]
ρ	transition rate from showing symptoms to recovering recovery	1/14 [1/days]	[9]
μ	transition rate to recovery for non-hospitalized	1/14 [1/days]	[9]
ϕ	productivity penalty for high-ability workers	0.7 [$\sim$]	Assumed
ι	productivity boost from immunity for high-ability workers	0.1 [$\sim$]	Assumed
ψ	productivity penalty for low-ability workers	0.5 [$\sim$]	Assumed
ξ	productivity boost from immunity for low-ability workers	0.2 [$\sim$]	Assumed
c_{h}	hospitalization costs for seniors	18,000 [USD]	[10]
c_{l}	hospitalization costs for non-seniors	12,000 [USD]	[10]
c_{d}	penalty of death on future economic output	25 [$\sim$]	Assumed
$y_{\mathrm{S,L}}$	average wage income for senior low-ability population	9,639.4 [USD]	[11]
$y_{\mathrm{S,H}}$	average wage income for senior high-ability population	28,062 [USD]	[11]
$y_{\mathrm{G,L}}$	average wage income for non-senior low-ability population	23,506.28 [USD]	[11]
$y_{\mathrm{G,H}}$	average wage income for non-senior high-ability population	70,077.59 [USD]	[11]
$\Pr(y_{\mathrm{S,L}})$	proportion of the population that is senior and low-ability	23.54%	[11]
$\Pr(y_{\mathrm{S,H}})$	proportion of the population that is senior and high-ability	9.53%	[11]
$\Pr(y_{\mathrm{G,L}})$	proportion of the population that is non-senior and low-ability	46.4%	[11]
$\Pr(y_{\mathrm{G,H}})$	proportion of the population that is non-senior and high-ability	20.53%	[11]
t_{l}	productivity penalty from length of isolation	0 [$\sim$]	Assumed
t_{h}	productivity penalty from length of isolation	0 [$\sim$]	Assumed

[$\sim$] denotes a dimensionless quantity.
Parameters used in the model, their descriptions, values, and their corresponding references.

S2 Table: Population transfer rates into and out of isolation, for the different scenarios.

**Parameter**	[1/days]			
	$\boldsymbol{\theta}$	$\boldsymbol{\theta}_{\text{Seniors}}$	κ	κ_{Seniors}
Baseline	$\mathbf{0}$	$\mathbf{0}$	0	0
Sudden release	$\mathbf{0}$	$\mathbf{0}$	0.03	0
Isolation re-enforcement	$[9\ 18\ 27\ 45\ 0]^{\mathrm{T}}$	$[4\ 8\ 12\ 20\ 0]^{\mathrm{T}}$	0	0
Gradual release	$[1\ 2\ 3\ 5\ 0]^{\mathrm{T}}$	$\mathbf{0}$	0.001	0
Weak restrictions on seniors	$[1\ 2\ 3\ 5\ 0]^{\mathrm{T}}$	$[0.0\ 0.2\ 0.3\ 0.4\ 0]^{\mathrm{T}}$	0.001	0.001

Parameter values used to regulate the population movement into and out of isolation. The baseline values (in black) are maintained during the entire simulation. The values used in the “sudden release of the population ...” scenario are marked in red. In this scenario, the baseline values of $\boldsymbol{\theta}$ and κ are maintained until the isolation policies are removed, then the values switch to those in “Sudden release”, afterwards when the isolation policies are re-enforced, the values switch to those in “Isolation re-enforcement. Lastly, the values used in the ”Progressive restart of the economy...” scenario are marked in blue. In this scenario as well, the baseline values are maintained until the isolation policies are lifted. Then, the values of $\boldsymbol{\theta}$ and κ switch to those marked in ”Gradual release”. The last row shows the values used to simulate the last scenario when the isolation of the senior population is not extreme.

S3 Table: Initial conditions for our simulations.

**Compartment**	Initial value [number of individuals]
Susceptible (S)	33,074,913
Isolated susceptible (Iso^S)	187,424,506
Exposed (E)	57,835
Isolated exposed (Iso^E)	327,734
Infected (I)	25,549
Hospitalized (H)	2,068
Isolated infected (Iso^I)	144,776
Dead (D)	14
Recovered (R)	6,393
Isolated recovered (Iso^R)	36,227
Susceptible seniors (S_S)	5,430,209
Isolated susceptible seniors (Iso_S^S)	103,173,979
Exposed seniors (E_S)	9,495
Isolated exposed seniors(Iso_S^E)	180,412
Infected seniors (I_S)	4,024
Hospitalized seniors (H_S)	6,224
Isolated infected seniors (Iso_S^I)	76,455
Dead seniors (D_S)	368
Recovered seniors (R_S)	940
Isolated recovered seniors (Iso_S^R)	17,876

The initial conditions used in our simulations. To obtain the initial conditions, we simulated our model with 33 initial infections and no isolation restrictions for five weeks. This simulates disease spread in the population before measures are taken by policymakers and individuals to limit its spread. The initial values reflect a relatively early stage of an epidemic, where disease has become widespread enough to make simple preventive and contract tracing measures inadequate, but where most of the population has not been infected.
Since our scenarios start with isolation policies already in place, we split the population obtained for every compartment into the non-isolated and isolated compartments. We allocate 85% and 95% of the non-seniors and seniors, respectively, under isolation.

References

[1] Wu Z, McGoogan JM. Characteristics of and important lessons from the coronavirus disease 2019 (COVID-19) outbreak in China: summary of a report of 72 314 cases from the Chinese Center for Disease Control and Prevention. Jama. 2020;.

[2] Lipsitch M, Donnelly CA, Fraser C, Blake IM, Cori A, Dorigatti I, et al. Potential biases in estimating absolute and relative case-fatality risks during outbreaks. PLoS neglected tropical diseases. 2015;9(7).

[3] Center for Health Protection; 2020. https://chp-dashboard.geodata.gov.hk/covid-19/en.html.

[4] CDC; 2020. https://www.cdc.gov/coronavirus/2019-ncov/covid-data/covidview/index.html.

[5] Zhou F, Yu T, Du R, Fan G, Liu Y, Liu Z, et al. Clinical course and risk factors for mortality of adult inpatients with COVID-19 in Wuhan, China: a retrospective cohort study. The Lancet. 2020;.

[6] Affairs H. American Hospital Capacity And Projected Need for COVID-19 Patient Care; 2020. *figshare* https://www.healthaffairs.org/do/10.1377/hblog20200317.457910/full/.

[7] Hellewell J, Abbott S, Gimma A, Bosse NI, Jarvis CI, Russell TW, et al. Feasibility of controlling COVID-19 outbreaks by isolation of cases and contacts. The Lancet Global Health. 2020;.

[8] Li R, Pei S, Chen B, Song Y, Zhang T, Yang W, et al. Substantial undocumented infection facilitates the rapid dissemination of novel coronavirus (SARS-CoV2). Science. 2020;.

[9] Pan F, Ye T, Sun P, Gui S, Liang B, Li L, et al. Time course of lung changes on chest CT during recovery from 2019 novel coronavirus (COVID-19) pneumonia. Radiology. 2020; p. 200370.

[10] healthsystemtracker. healthsystemtracker; 2020. *figshare* https://www.healthsystemtracker.org.

[11] Ruggles S, Flood S, Goeken R, Grover J, Meyer E, Pacas J, et al. IPUMS USA: Version 8.0 [dataset]. Minneapolis, MN: IPUMS. 2018;10:D010.
