# Supplementary material for: Public policy and economic dynamics of COVID-19 spread: A mathematical modeling study
Source: PLoS One. 2020 Dec 22;15(12):e0244174. doi: 10.1371/journal.pone.0244174 (PMC7755180; doi:10.1371/journal.pone.0244174)
Supplement: S1 Fig — This figure shows the complete results of the baseline simulation. It includes plots of the number of individuals in each compartment as a function of time. (PDF) [file pone.0244174.s003.pdf]

S1 Fig. Complete simulation for the baseline scenario

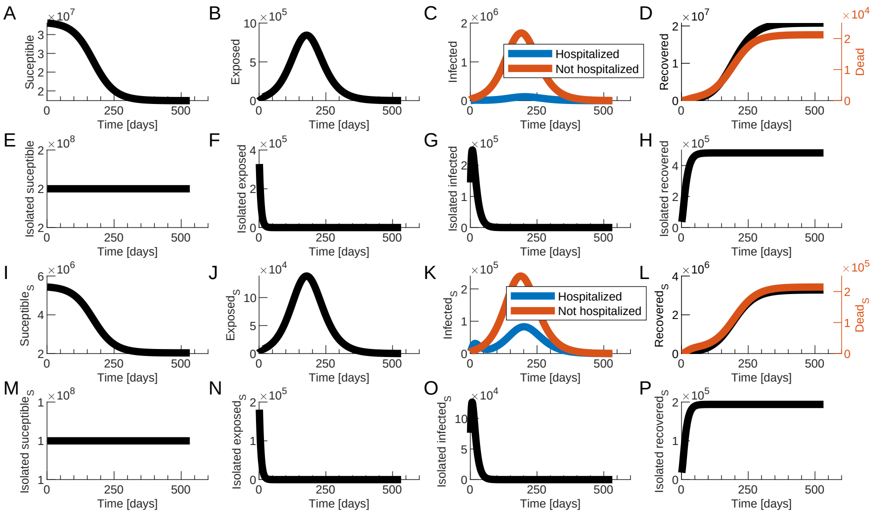

Figure 1: **Compartments in the baseline scenario.** (**A**) Number of non-senior individuals in the susceptible compartment. (**B**) Number of non-senior individuals in the exposed compartment. These individuals are not contagious and will become infected. (**C**) Number of non-senior individuals in the infected compartments. The red curve shows the number of infected, the blue curve represents those that are hospitalized. (**D**) Number of non-senior individuals that have recovered or passed away due to COVID-19. (**E**) Number of non-senior susceptible individuals in isolation. (**F**) Number of non-senior exposed individuals in isolation. (**G**) Number of non-senior infected individuals in isolation. (**H**) Number of non-senior individuals in isolation that have recovered from infection. (**I-P**) Same compartments as (**A-H**) for the senior population.
